# Delayed Spinal Arachnoiditis Following Aneurysmal Subarachnoid Hemorrhage: A Case Report

**DOI:** 10.7759/cureus.2031

**Published:** 2018-01-06

**Authors:** Rimal H Dossani, Devi P Patra, Hai Sun, Anil Nanda, Hugo Cuellar

**Affiliations:** 1 Department of Neurosurgery, LSU Health Sciences Center Shreveport

**Keywords:** spinal arachnoiditis, spinal arachnoid cyst, subarachnoid hemorrhage

## Abstract

Spinal arachnoiditis (SA) is a rare and delayed complication of aneurysmal subarachnoid hemorrhage (aSAH). We present a case of delayed SA associated with thoracic and lumbar arachnoid cysts in a patient with aSAH secondary to a ruptured vertebral artery aneurysm. The patient underwent a thoracic laminectomy for decompression of the spinal cord, lysis of arachnoid adhesions, and fenestration of an arachnoid cyst. We present the pathogenesis, diagnosis, treatment, and management of spinal arachnoiditis as a rare complication of aSAH.

## Introduction

Spinal arachnoiditis (SA) with associated arachnoid cyst is a rare and delayed complication of aneurysmal subarachnoid hemorrhage (aSAH). Although the exact mechanism of SA following aSAH is unknown, it is believed that hemolyzed blood products provoke an inflammatory response in the subarachnoid space, which leads to scarring of the leptomeninges and subsequent arachnoiditis [1]. The most common location of SA is the thoracic spine, and therefore, the majority of symptomatic patients with SA present with spastic lower extremity weakness and bladder dysfunction [1]. Treatments discussed in the literature range from observation to microscopic lysis of arachnoid adhesions to syrinx-subarachnoidal shunting [2-3]. We present a case of a patient who developed delayed SA and arachnoid cyst following aSAH from rupture of a posterior circulation aneurysm. The presentation, imaging, and treatment of the patient are described. A literature review of SA following SAH is presented in the Discussion.   

## Case presentation

**Figure 1:**
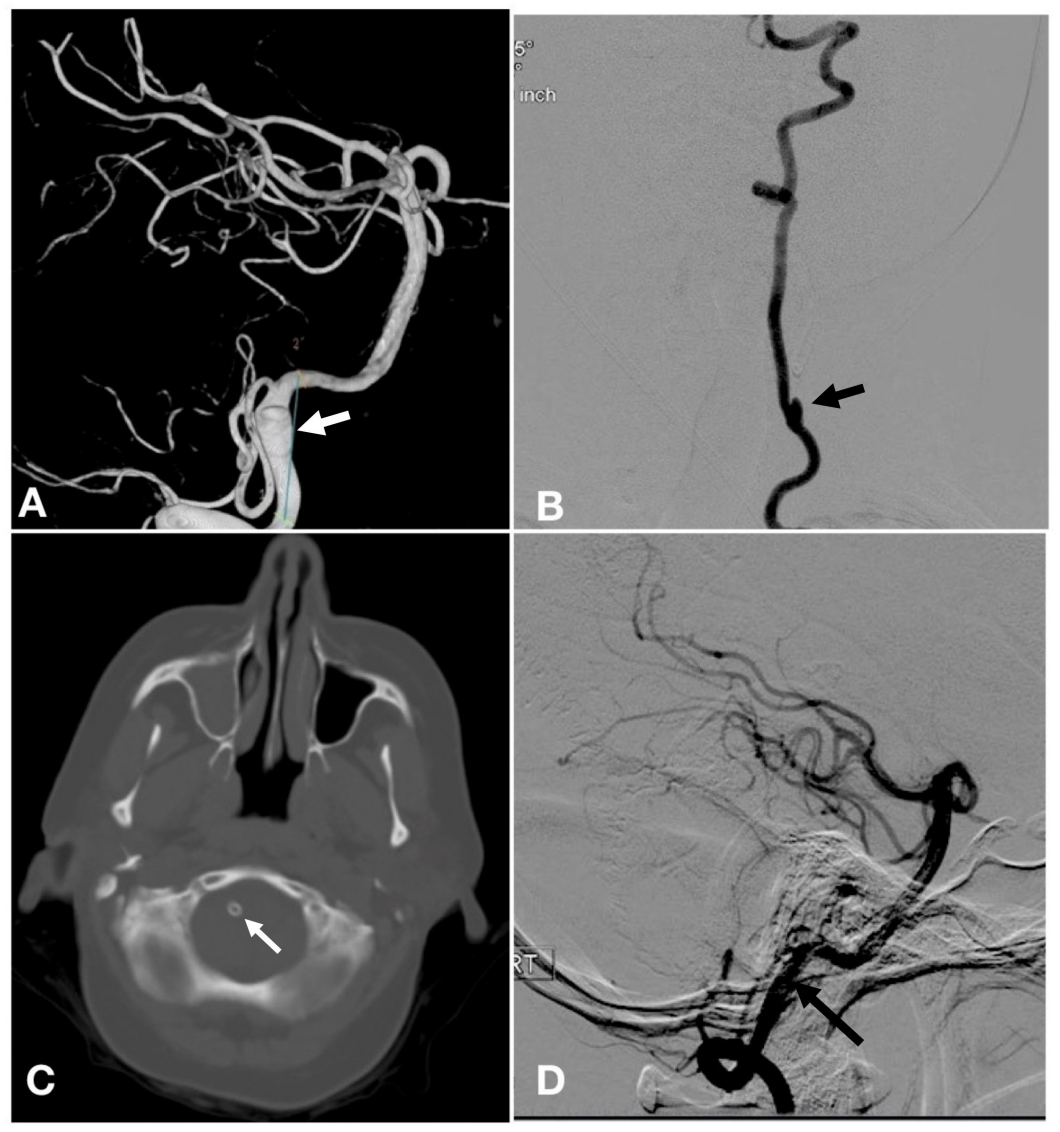
Cerebral arteriogram and noncontrast computed tomography CT head demonstrating right-sided intracranial vertebral artery aneurysm treated with Pipeline embolization device. (A) Three dimensional reconstruction of right vertebral artery injection showing fusiform aneurysm (arrow) proximal to the origin of the posterior inferior cerebellar artery; (B) anteroposterior injection of the left vertebral artery showing dissecting aneurysm (arrow) at the C5 level; (C) CT head without contrast showing flow diversion with Pipeline (arrow) in right vertebral artery; (D) Follow-up right vertebral artery injection seven months after flow diversion showed complete remodeling of vertebral artery and resolution of aneurysm (arrow).

The patient is a 43-year-old female who presented one month after developing subarachnoid hemorrhage (SAH). Her symptoms at the time of SAH were a sudden-onset headache and bilateral eye blindness from vitreous hemorrhage (Terson syndrome). Upon presentation at our facility, the patient had no headache, was alert, and had 5/5 strength in all her extremities, but her vision was limited to light perception only. Computed tomography (CT) scan of the head without contrast did not show residual subarachnoid hemorrhage or hydrocephalus. Because of her history of SAH with thunderclap headache, a cerebral arteriogram was performed, which demonstrated a right-sided, fusiform, V4 segment vertebral artery aneurysm proximal to the origin of the posterior inferior cerebellar artery (PICA) (Figure 1A) and a left-sided, dissecting, cervical vertebral artery aneurysm at the level of C5 (Figure 1B). Flow diversion for right-sided, V4 segment aneurysm was performed (Figure 1C). The patient returned seven months later for follow-up angiogram, which showed complete remodeling of the vertebral artery and resolution of fusiform, intracranial aneurysm (Figure 1D). During the same procedure, the patient underwent flow diversion of the left-sided, cervical vertebral artery aneurysm. One year after initial presentation with SAH, the patient presented with bilateral lower extremity weakness (3/5 strength) with overflow bladder incontinence.

**Figure 2:**
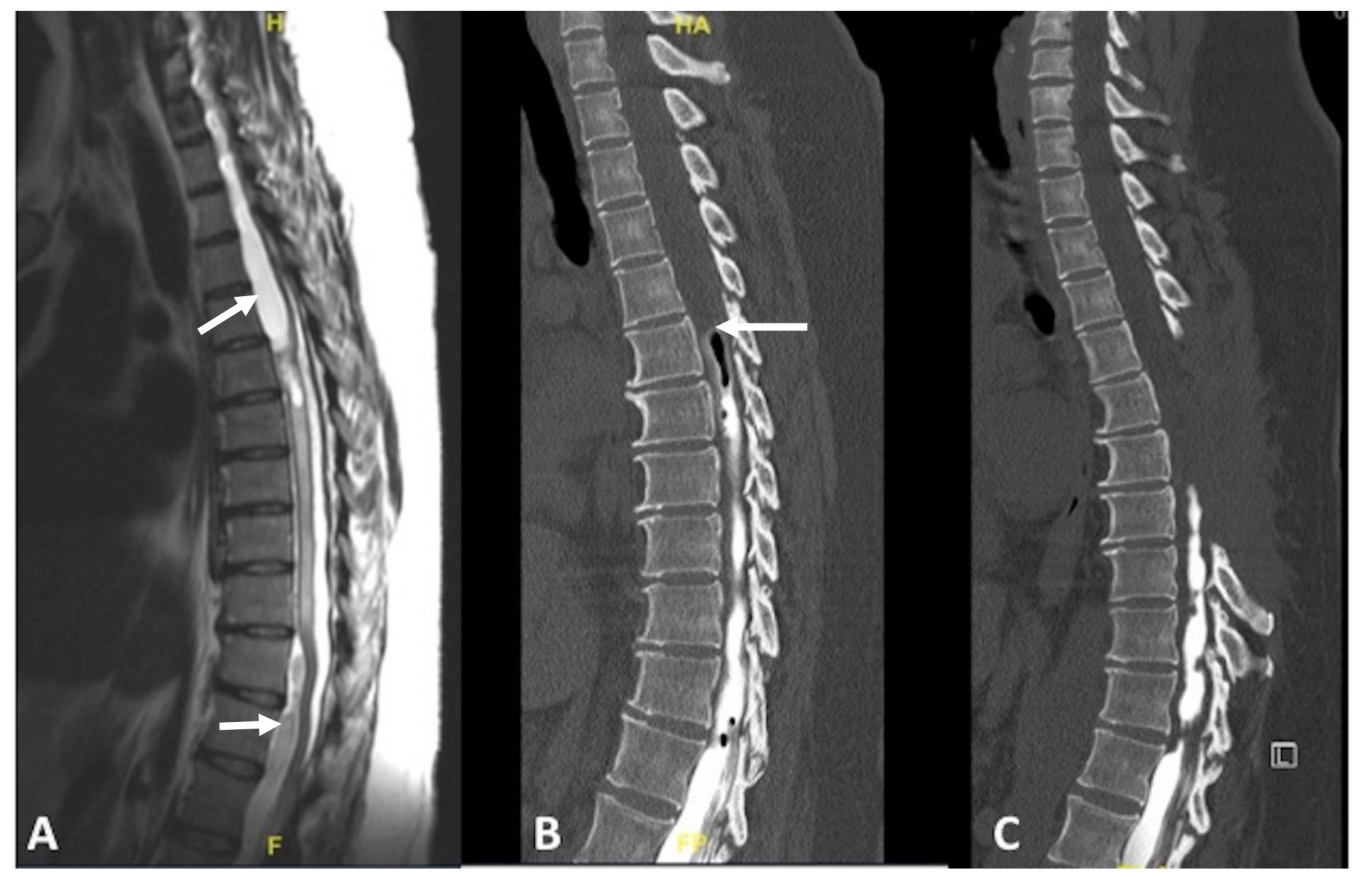
T2-weighted MRI and CT myelogram demonstrating spinal arachnoiditis with associated arachnoid cysts. (A) T2-weighted sagittal MRI without contrast showing arachnoid cysts (arrows) ventral to the thoracic and lumbar spinal cord with cord compression. The spinal cord is ventrally displaced at the T5-8 levels due to arachnoiditis in the dorsal leptomeninges; (B) CT myelogram sagittal view showing stagnation of contrast at the T5 level (arrow), a sign that arachnoiditis is blocking cerebrospinal fluid flow; (C) Postoperative myelogram showing T5-8 laminectomy with no improvement in cerebrospinal fluid flow. MRI: magnetic resonance imaging; CT: computed tomography

T2-weighted magnetic resonance imaging (MRI) of the thoracolumbar spine demonstrated an irregular cerebrospinal fluid (CSF) signal from T5-8 around the spinal cord with multiloculated cysts (Figure 2A). An arachnoid cyst, located ventral to the thoracic and lumbar spinal cord, was noted to cause the spinal cord compression. A CT myelogram showed an abrupt stop in the cerebrospinal fluid flow at the level of T5 (Figure 2B). The patient underwent a T5-8 laminectomy for decompression of the spinal cord, lysis of arachnoid adhesions, and fenestration of the arachnoid cyst. The spinal cord was noted to be decompressed at the end of the procedure, and the dura was closed in a watertight fashion.

Postoperatively, the patient’s strength improved to 4/5 in both lower extremities. However, the patient returned three months later with recurrence of the arachnoiditis and cysts from T5-8. She developed complete lower extremity paralysis (0/5 strength) with loss of bladder control. Repeat CT scan of the head did not show hydrocephalus. Repeat CT myelogram showed no improvement in the contrast flow above the T8 level (Figure 2C). No further surgical treatment was performed, and the patient was sent for physical rehabilitation. Upon follow-up one year after her spinal operation, the patient remained completely paralyzed in her lower extremities (0/5 strength). 

## Discussion

The patient presented in this report developed delayed lower extremity weakness, hyperreflexia, and loss of bladder control one year after subarachnoid hemorrhage (SAH) secondary to a ruptured V4 segment vertebral artery aneurysm. Although the patient improved transiently after surgical decompression, lysis of adhesions, and fenestration of arachnoid cysts, she returned with worsening lower extremity weakness and loss of bladder control.

The etiology of SA following SAH is debatable. Some speculate that the heightened inflammatory microenvironment of the subarachnoid space following SAH incites a fibroproliferative process leading to SA [1]. The exact incidence of SA following SAH is unknown, but it is reasonable to assume that symptomatic SA following SAH is exceedingly rare, as less than 20 cases are reported in peer-reviewed literature. Since all patients with SAH should theoretically have an inflammatory response to hemolyzed blood products circulating throughout the subarachnoid space, it is intriguing why such a small number of patients go on to develop symptomatic SA. It is possible that subclinical SA is more common but is not reported because such cases never receive clinical attention. A study performed by Shaw, et al. looking at patients with SA from causes other than SAH noted that only 25% of patients with SA were symptomatic [4].

Kok, et al. presented two cases with symptomatic SA following SAH, both of whom developed posthemorrhagic hydrocephalus and subsequent lower extremity weakness [5]. One of the patients underwent shunting for hydrocephalus and her lower extremity hyperreflexia and weakness improved over time. However, the other patient improved with observation alone without treatment of hydrocephalus. In a thorough literature review, the authors reviewed 14 cases to highlight factors that may predispose patients to develop symptomatic SA following SAH. All but one patient had SA and arachnoid cysts in the thoracic spine, which may be due to pooling of blood products in the kyphotic segment of the dependent thoracic spine. Therefore, patients with prolonged bed rest following SAH may be predisposed to developing SA. None of the patients had aneurysms of the anterior circulation, while six patients had posterior circulation aneurysms. Delayed, post-hemorrhagic hydrocephalus may play a role in the development of SA, as reported by Kibler, et al., who showed autopsy findings of adhesions and thickening of the leptomeninges in a patient who died from communicating hydrocephalus 10 months after SAH [6].

The treatments discussed in the literature range from observation to surgical decompression of the arachnoid cyst to syrinx-subarachnoidal shunting. Tumialan, et al. reported improvement in lower extremity symptoms following thoracic laminectomy for lysis of arachnoid adhesions in a patient with SA secondary to SAH [1]. Ginanneschi, et al. reported a recurrence of an arachnoid cyst (secondary to SAH) of the same size two weeks after removal through hemilaminectomy and recommended that conservative management with observation is the best treatment choice [2]. Iwatsuki, et al. performed laminectomies on two patients (no history of SAH) with syringomyelia associated with spinal arachnoiditis for lysis of adhesions and syrinx-subarachnoidal shunting to a location distal to the zone of arachnoiditis [3]. Both patients improved postoperatively after a short course of physical rehabilitation. Our patient, however, had an aggressive course of SA that was refractory to surgical treatment.

## Conclusions

Spinal arachnoiditis (SA) is a rare, delayed complication following aneurysmal subarachnoid hemorrhage (aSAH), occurring more commonly in patients with ruptured posterior circulation aneurysms. The patient in this case report had an aSAH secondary to a ruptured vertebral artery aneurysm. One year following aSAH, she developed bilateral lower extremity weakness (4/5 strength), and her spinal MRI demonstrated thoracic SA with associated arachnoid cysts. The patient underwent thoracic laminectomy for decompression of spinal cord, lysis of adhesions and fenestration of arachnoid cysts. The patient's lower extremity strength improved transiently during the postoperative period but worsened to complete lower extremity paraplegia at her last clinic follow-up. CT myelogram performed at her last follow-up did not show improvement in cerebrospinal fluid flow in the thoracic region where the laminectomy had been performed. No further surgical intervention was performed. The literature offers several management strategies for SA secondary to aSAH, ranging from observation to spinal decompression and lysis of adhesions to syrinx-subarachnoid shunting. There is no standardized treatment guideline at this time, and surgeons are advised to treat each patient on a case-by-case basis.
